# Activation of Kv7 Potassium Channels Inhibits Intracellular Ca^2+^ Increases Triggered By TRPV1-Mediated Pain-Inducing Stimuli in F11 Immortalized Sensory Neurons

**DOI:** 10.3390/ijms20184322

**Published:** 2019-09-04

**Authors:** Paolo Ambrosino, Maria Virginia Soldovieri, Erika Di Zazzo, Gianluca Paventi, Fabio Arturo Iannotti, Ilaria Mosca, Francesco Miceli, Cristina Franco, Lorella Maria Teresa Canzoniero, Maurizio Taglialatela

**Affiliations:** 1 Department of Science and Technology, University of Sannio, 82100 Benevento, Italy; 2 Department of Medicine and Health Sciences “V. Tiberio”, University of Molise, 86100 Campobasso, Italy; 3 Institute of Biomolecular Chemistry, National Research Council, Pozzuoli, 80121 Naples, Italy; 4 Division of Pharmacology, Department of Neuroscience, University of Naples “Federico II”, 80131 Naples, Italy

**Keywords:** F11 cells, retigabine, XE991, capsaicin, bradykinin

## Abstract

Kv7.2-Kv7.5 channels mediate the M-current (I_KM_), a K^+^-selective current regulating neuronal excitability and representing an attractive target for pharmacological therapy against hyperexcitability diseases such as pain. Kv7 channels interact functionally with transient receptor potential vanilloid 1 (TRPV1) channels activated by endogenous and/or exogenous pain-inducing substances, such as bradykinin (BK) or capsaicin (CAP), respectively; however, whether Kv7 channels of specific molecular composition provide a dominant contribution in BK- or CAP-evoked responses is yet unknown. To this aim, Kv7 transcripts expression and function were assessed in F11 immortalized sensorial neurons, a cellular model widely used to assess nociceptive molecular mechanisms. In these cells, the effects of the pan-Kv7 activator retigabine were investigated, as well as the effects of ICA-27243 and (S)-1, two Kv7 activators acting preferentially on Kv7.2/Kv7.3 and Kv7.4/Kv7.5 channels, respectively, on BK- and CAP-induced changes in intracellular Ca^2+^ concentrations ([Ca^2+^]_i_). The results obtained revealed the expression of transcripts of all *Kv7* genes, leading to an I_KM_-like current. Moreover, all tested Kv7 openers inhibited BK- and CAP-induced responses by a similar extent (~60%); at least for BK-induced Ca^2+^ responses, the potency of retigabine (IC_50_~1 µM) was higher than that of ICA-27243 (IC_50_~5 µM) and (S)-1 (IC_50_~7 µM). Altogether, these results suggest that I_KM_ activation effectively counteracts the cellular processes triggered by TRPV1-mediated pain-inducing stimuli, and highlight a possible critical contribution of Kv7.4 subunits.

## 1. Introduction

The Kv7 (KCNQ) subfamily of voltage-gated K^+^ channel subunits includes five members (from Kv7.1 to Kv7.5), each showing specific tissue distribution and functional roles [1]. While Kv7.1 subunits are mainly distributed in cardiac cells [2], all other Kv7 subunits are expressed in neuronal cells, where they represent the molecular basis for the M-current (I_KM_) [3,4,5], a repolarizing K^+^-selective current playing a pivotal role in excitability control [6]. Analogously to other voltage-gated K^+^ channels subunits, Kv7 subunits show a topological arrangement in which a core domain with six transmembrane segments (S_1_ to S_6_) is flanked by intracellularly located N- and C-*termini*. Within the core domain, the first four transmembrane segments (S_1_ to S_4_) form the voltage sensing domain (VSD), while S_5_, S_6_, and the intervening linker contribute to the formation of the pore region and selectivity filter; the N- and C-*termini* provide binding sites for several molecules critically influencing channel assembly, regulation, subcellular trafficking, and function [7,8,9,10,11,12]. Functional Kv7 channels assemble as tetramers of identical (homomers) or compatible (heteromers) subunits [1,5]; therefore, a large number of Kv7 channels of different molecular composition can be assembled in each individual cells at specific developmental stages, a phenomenon likely contributing to the adaptation of cellular responses to changes in environmental conditions or pathophysiological events [13,14].

Due to their role in neuronal excitability control, Kv7 channels are currently regarded as critical players in hyperexcitability diseases such as epilepsy, mania, ADHD (Attention-Deficit/Hyperactivity Disorder), addiction to psychostimulants, and depression. Moreover, neuronal hyperexcitability also plays a critical pathogenetic role in the development of pain states accompanying nerve crushing, inflammatory states, central, and/or peripheral neuropathies, also when the latter are caused by exposure to clinically-used drugs such as antineoplastics [15,16]. As a matter of fact, the pan-Kv7 channel opener flupirtine is effective in several animal models of nociception [17,18], and has been used clinically as a non-opioid analgesic in humans, before being withdrawn from the market in 2018 because of severe drug-induced hepatotoxic responses. Consistent with their prominent role in pain states, Kv7 channels are expressed in nociceptive neurons of the ganglia and of the spinal cord; their inhibition mediates a significant part of the nociceptive effects of inflammatory and pain-inducing mediators such as exogenous proteases and bradykinin (BK) [19,20]. A decreased expression of Kv7 channels has been shown to contribute to neuropathic hyperalgesia following partial sciatic nerve ligation in rats [13]; moreover, drug-induced activation of Kv7 channels inhibit C and Aδ fiber-mediated responses of dorsal horn neurons evoked by natural or electrical afferent stimulation [21] and counteracts receptor-induced M current inhibition in nociceptors during inflammatory pain [22]. Intriguingly, Kv7 channels are also present on axons of unmyelinated, including nociceptive, peripheral human nerve fibers: activation of these channels reduces the ectopic generation of action potentials occurring during neuropathic pain states [23]. Notably, a functional interaction has been demonstrated between Kv7 and transient receptor potential vanilloid 1 (TRPV1) channels [24], expressed predominantly in peripheral nociceptors [25] and activated by TRPV1-mediated pain-inducing substances such as the endogenous inflammatory mediator BK or the exogenous burning agent capsaicin (CAP).

However, whether Kv7 channels of specific molecular composition provide a dominant contribution in BK- or CAP-evoked responses is yet unknown. To answer this question, in the present study, we have first confirmed the expression of transcripts encoding for all Kv7 members and functional I_KM_ in mouse neuroblastoma/rat dorsal root ganglionic hybrid neurons (F11 cells) [26], a cellular model widely used to assess nociceptive molecular mechanisms in which the expression and function of TRPV1 channels has been previously reported [27]. In these cells, Ca^2+^-imaging experiments were subsequently performed to assess the possible participation of Kv7 channels in the changes of intracellular Ca^2+^ concentrations ([Ca^2+^]_i_) triggered by BK or CAP; to this aim, the ability of the pan-Kv7 activator retigabine (RTG), a structural analogue of flupirtine [15,18], in inhibiting BK- and CAP-evoked [Ca^2+^]_i_ responses in F11 cells was compared to that of ICA-27243, a relatively selective Kv7.2/Kv7.3 opener [28], and of (S)-1, a relatively selective Kv7.4/Kv7.5 opener [29].

The results obtained confirmed that Kv7.2, Kv7.3, and Kv7.4 transcripts are expressed in differentiated F11 cells, where a current having the biophysical and pharmacological characteristics of an M-current can be functionally identified. Moreover, all three tested Kv7 openers reduced BK- and CAP-induced [Ca^2+^]_i_ responses, an effect fully reversed by co-exposure to the selective Kv7 blocker XE991 [30]. In particular, the potency of RTG was higher than that of ICA-27243 or (S)-1, whereas the maximal inhibitory effect was similar among all three drugs. Collectively, the present results show that drug-induced activation of Kv7 channels can significantly inhibit cellular responses underlying pain sensation, confirming the role of this subfamily of K^+^ channels as critical target for novel pain treatment strategies; pharmacological data also suggest a possible, previously-unrecognized, contribution of channels containing Kv7.4 subunits in blunting [Ca^2+^]_i_ rises triggered by TRPV1-dependent endogenous and/or exogenous pain-inducing stimuli.

## 2. Results

### 2.1. Biochemical and Functional Evidence for Kv7 Expression in F11 Cells

F11 cells, a hybrid cell line obtained from the fusion of differentiated mouse neuroblastoma cells and primary cells from rat dorsal root ganglia, are widely used to investigate the molecular mechanisms triggered by nociceptive stimuli. In fact, these cells express both neuronal (α-tubulin III, NF-160, and NeuN) and nociceptive (delta-opioid, prostaglandin, and BK receptors) markers [31], which display the strongest [Ca^2+^]_i_ responses to BK among neuronal cell lines [32], and synthesize and release a substance P-like compound [33,34]. When differentiated (see the *Methods* section for details), F11 cells exhibit a mature neuronal morphology, with long neurites and an increased expression of BK receptors and voltage-gated calcium channels (VGCCs) [33]. Moreover, transient [Ca^2+^]_i_ increases were observed upon exposure not only to BK, but also CAP in F11 cells, both being markedly reduced by TRPV1 antagonists [27].

**Figure 1:**
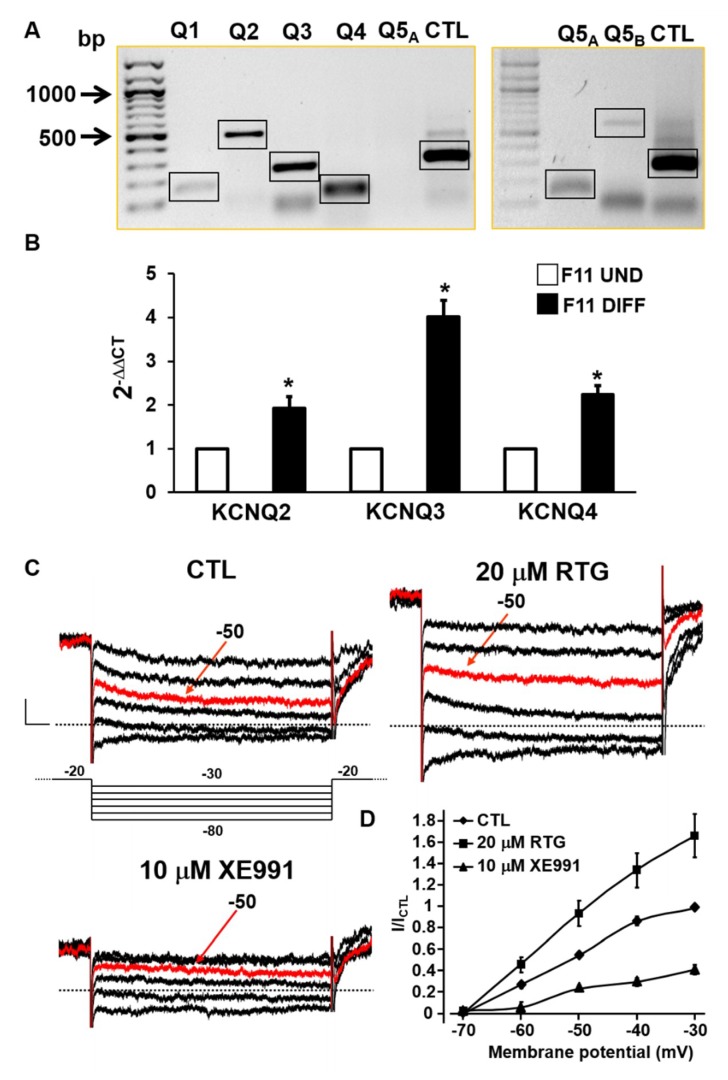
Biochemical and functional evidence of Kv7 expression in F11 cells. (**A**) Representative images of electrophoretic separations of RT-PCR reactions using total RNA extracted from undifferentiated F11 cells and primers designed on conserved sequence regions of KCNQ1 (Q1), KCNQ2 (Q2), KCNQ3 (Q3), KCNQ4 (Q4), KCNQ5 (Q5) mRNAs of both mouse and rat genes. Arrows indicate the molecular mass of DNA marker. “A” and “B” in subscripts indicate two distinct primer pairs designed on distinct regions of KCNQ5 mRNA; the housekeeping *GAPDH* gene was used as control (CTL). Black boxes indicate expected amplicons for each reaction. The image is representative of data obtained from three separate experiments. (**B**) Quantification of KCNQ mRNAs detected in real-time PCR performed using primers for *KCNQ2*, *KCNQ3* or *KCNQ4* genes, as indicated, from total RNAs extracted from undifferentiated (white bars) or differentiated (black bars) F11 cells. Asterisks indicate values significantly different (*p* < 0.05) versus respective controls. cDNAs samples were amplified in triplicate in each one-assay run (*n* = 4). (**C**) Representative current traces recorded from differentiated F11 cells upon the application to the voltage protocol shown below the first set of traces, in control solution (CTL) or upon exposure to the indicated drugs. Red traces correspond to currents recorded at −50 mV in all tested conditions. (**D**) Quantification (*n* = 3–5) of currents measured in cells recorded as in C and expressed as % of currents measured in the same cell at −30 mV before drug exposure.

To investigate the possible role of Kv7 channels in TRPV1-activating pain-inducing stimuli, the expression of all *KCNQ* genes in undifferentiated F11 cells was first analyzed. As shown in the left panel of Figure 1A, semi-quantitative RT-PCR experiments revealed the presence of amplicons of the expected size for KCNQ2 (489 bp), KCNQ3 (238 bp), or KCNQ4 (98 bp) mRNAs, as previously reported in dorsal root ganglion neurons (DRG) [21], whereas the amplicons for KCNQ1 (143 bp) and KCNQ5 (99 bp) were very faint or undetectable, respectively. Since KCNQ5 mRNA expression has been previously described in rat DRGs [21], more stringent experimental conditions were used to reveal KCNQ5-specific transcripts; as a matter of fact, when 5 µg (instead of 1 µg) of total RNA in the retrotranscription reaction were used, a KCNQ5-specific band was clearly detected, as also confirmed by the results obtained with a second primer pair (amplicon expected size of 564 bp) directed toward a different region of KCNQ5 mRNA (right panel of Figure 1A). Given the lower intensity of the bands corresponding to KCNQ1 and KCNQ5 mRNAs when compared to that of the other Kv7 family members, quantitative PCR experiments were subsequently performed to investigate KCNQ2, KCNQ3, and KCNQ4 transcript expression in control and differentiated F11 cells. As reported in Figure 1B, all tested KCNQ mRNAs were detected in undifferentiated F11 cells, with KCNQ4 being the most abundant (Ct ~ 25), followed by KCNQ2 (Ct ~ 28) and KCNQ3 (Ct ~ 34); transcripts encoding for all three Kv7 subunits were significantly increased upon F11 cell differentiation.

Given these results, perforated patch-clamp recordings were performed to investigate whether a functional I_KM_ was detectable in differentiated F11 cells; to this aim, a standard voltage protocol was adopted in which cells were held at a steady depolarized potential sufficient to activate the M-current, followed by hyperpolarizing voltage steps to reveal I_KM_ deactivation (see the Methods section for details). Using this protocol, the contamination by other voltage-gated currents is minimized since these are largely inactivated at the depolarizing holding voltage, while the contribution of non-inactivating currents such as I_KM_ is maximized [35]. As shown in Figure 1C, this protocol allowed the identification of a slowly-deactivating current corresponding to the I_KM_ tails during the hyperpolarizing steps. The observation that these currents were significantly potentiated upon exposure to the Kv7 activator RTG (20 µM) [15] and inhibited by the Kv7 blocker XE991 (10 µM) [30] (Figure 1C,D) strongly suggests their molecular identity as Kv7-mediated I_KM_.

### 2.2. Kv7 Activators Reduce BK-evoked [Ca^2+^]_i_ Responses in F11 Cells

**Figure 2:**
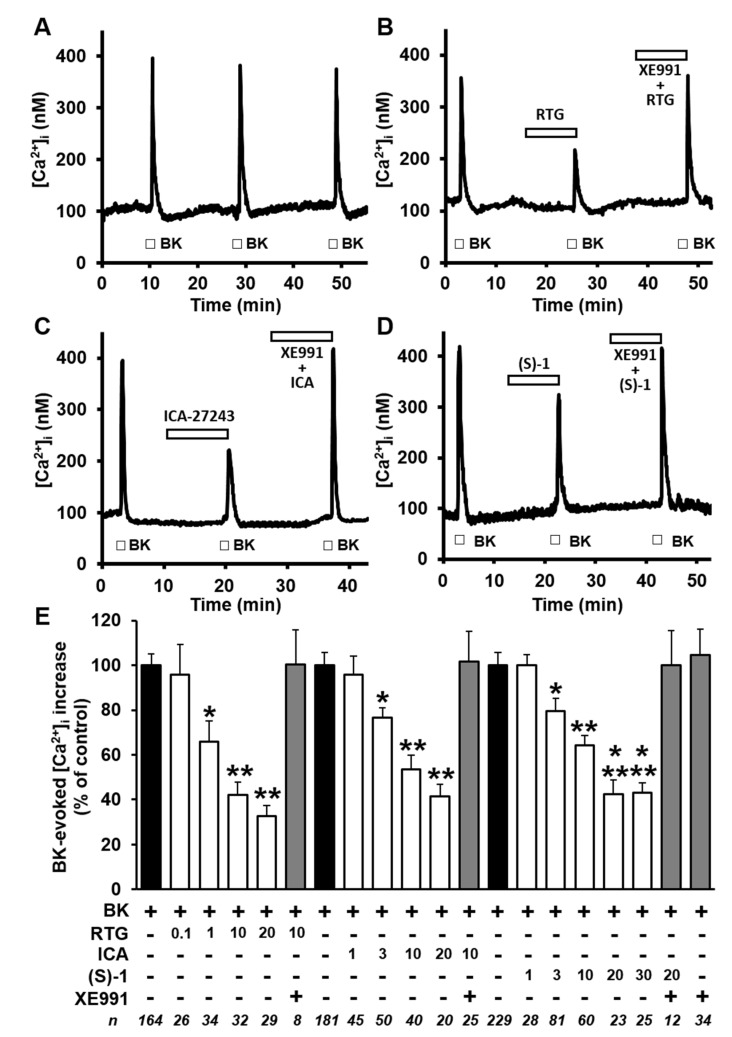
Effects of Kv7 modulators on bradykinin (BK)-induced [Ca^2+^]_i_ responses in differentiated F11 cells. (**A**–**D**) Representative traces showing the effect of three subsequent exposures to BK (250 nM) on [Ca^2+^]_i_ in differentiated F11 cells in control conditions (**A**) or after exposure to 10 µM of the indicated drugs (**B**–**D**). The length of the bars indicates the duration of each drug exposure. (**E**) Quantification of the effects of the indicated drugs (whose concentrations are reported in µM at the bottom of the panel) on the BK-induced [Ca^2+^]_i_ responses. Data are expressed as percent of [Ca^2+^]_i_ increase prompted by the first BK exposure. Each bar is the mean ± SEM of separate determinations (indicated as n values at the bottom of the panel) performed in at least three experimental sessions. *, **, or *** = *p* < 0.05 versus the immediately lower concentrations tested of the same drug.

Having found a significant expression and function of Kv7 channels in differentiated F11 cells, their possible role in the modulation of BK-induced [Ca^2+^]_i_ responses was then investigated. As already reported [27,36] and shown here in Figure 2A, three subsequent 20-s exposures to BK (250 nM) applied every 20 min induced identical transient increases in [Ca^2+^]_i_ in about 84% (574/682) of cells tested. BK-triggered [Ca^2+^]_i_ increases were mostly dependent on extracellular Ca^2+^ influx and were also largely prevented upon exposure to the canonical TRPV1 antagonist capsazepine (CPZ), thus confirming a critical role for TRPV1 channels in BK-induced [Ca^2+^]_i_ responses [27]. BK-induced [Ca^2+^]_i_ increases were dose-dependently reduced by the pan-Kv7 opener RTG (0.1–20 µM; Figure 2B–E), showing an IC_50_ of 1.04 ± 0.47 µM. In addition, the Kv7 blocker XE991 (10 µM) was unable *per se* to modify both resting [Ca^2+^]_i_ and BK-triggered [Ca^2+^]_i_ responses, but completely reversed the inhibitory effects of RTG on BK-induced [Ca^2+^]_i_ increases (Figure 2B–E).

As previously introduced, RTG is a pan-Kv7 activator with little selectivity among channels formed by distinct combinations of Kv7 subunits. In attempt to dissect the contribution played by channels formed by specific Kv7 subunits in the observed functional effects of RTG, the effects of ICA-27243 [28], a benzamide derivative provided of a certain degree of selectivity for Kv7.2/Kv7.3 channels [37], were also investigated. On the other hand, to verify the possible contribution of heteromeric channels composed of Kv7.4/Kv7.5 subunits, the rather selective Kv7.4/Kv7.5 activator (S)-1 [29], was also investigated. Exposure of F11 cells to ICA-27243 (1–20 µM; Figure 2C–E) or to (S)-1 (1–30 µM; Figure 2D–E) dose-dependently counteracted BK-induced [Ca^2+^]_i_ rises, with a maximal efficacy identical to that of RTG (~ 60% maximal blockade), although their potency was lower than that of RTG (IC_50_s were 5.3 ± 1.9 µM and 7.7 ± 5.6 µM for ICA-27243 and (S)-1, respectively; *p* < 0.05 versus RTG; *p* > 0.05 between ICA-27243 and (S)-1).

### 2.3. Kv7 Activators Reduce CAP-evoked [Ca^2+^]_i_ Responses in F11 Cells

**Figure 3:**
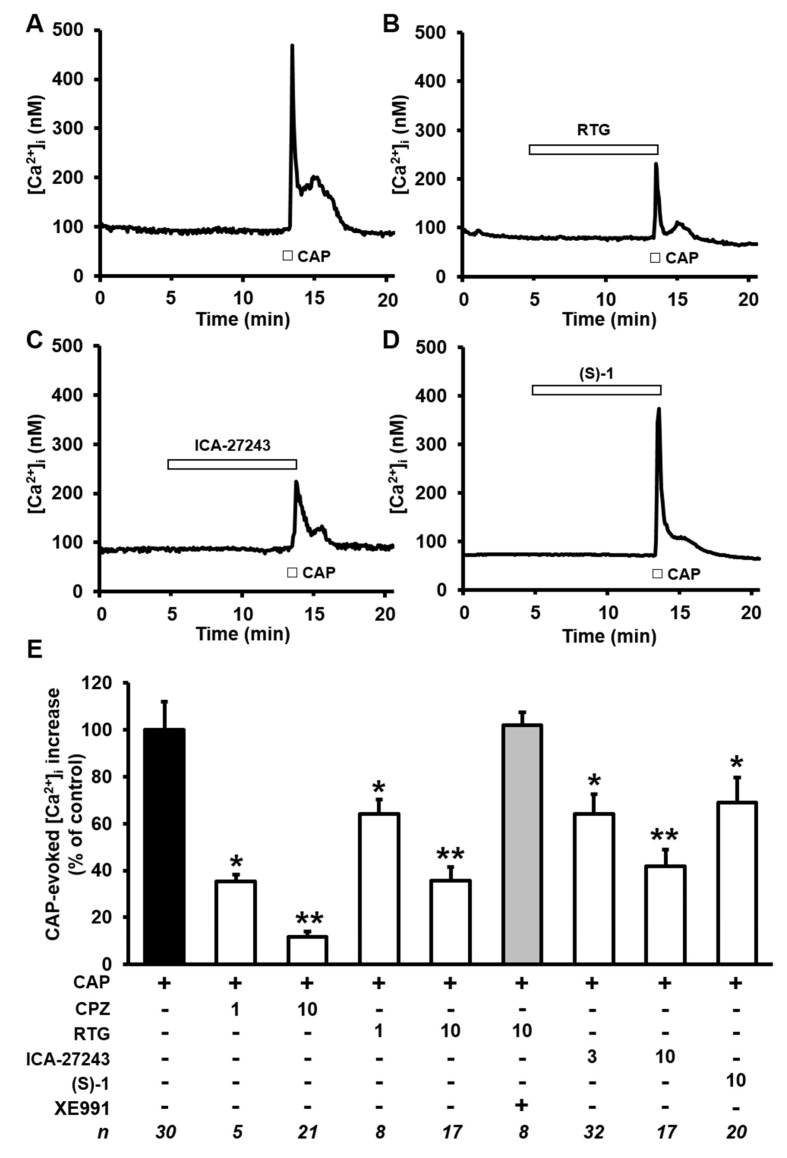
Effects of Kv7 modulators on capsaicin (CAP)-induced [Ca^2+^]_i_ responses in differentiated F11 cells. (**A**–**D**) Representative traces showing the effect of a single exposure to CAP (50 µM) on [Ca^2+^]_i_ in F11 cells incubated in control conditions (**A**) or after exposure to 10 µM of the indicated drugs (**B–D**). The length of the bars indicates the duration of each drug exposure. (**E**) Quantification of the effects of the indicated drugs (whose concentrations are reported in µM at the bottom of the panel) on CAP-induced [Ca^2+^]_i_ responses. Data are expressed as percent of CAP-induced [Ca^2+^]_i_ increases in control solution. Each bar is the mean ± SEM of separate determinations (indicated as n values at the bottom of this panel) performed in at least three experimental sessions. * or ** = *p* < 0.05 versus the immediately lower concentrations tested of the same drug.

To verify whether Kv7 subunits activation also opposes [Ca^2+^]_i_ rises triggered by direct-acting TRPV1 agonists, the effects of Kv7 activators in F11 cells exposed to the canonical TRPV1 agonist CAP [38] were also investigated (Figure 3). As previously reported [27], the kinetics of [Ca^2+^]_i_ changes induced by CAP were markedly different from those elicited by BK: in fact, although [Ca^2+^]_i_ increases were similarly fast, [Ca^2+^]_i_ decay after *stimulus* removal was markedly slower for CAP, causing [Ca^2+^]_i_ to remain higher than basal for several minutes (Figure 3A) and triggering strong desensitization of the TRPV1 channels, such that multiple CAP applications failed to trigger [Ca^2+^]_i_ responses of similar amplitude (as in the case of BK). Because of these differences in the kinetics of TRPV1 agonist-induced [Ca^2+^]_i_ response, a protocol in which each cell was challenged with a single CAP exposure, both in control condition or after exposure for 7–8 min to the Kv7 agonists. CAP-induced [Ca^2+^]_i_ rises were significantly blocked upon co-exposure to the TRPV1 antagonist CPZ, when used at a concentration (1 µM) and application times (<1 min) that do not affect VGCCs in DRG neurons [39]; as expected, higher concentrations of the canonical TRPV1-antagonist CPZ (10 µM) almost completely (>90%) inhibited CAP-induced [Ca^2+^]_i_ changes (Figure 3E). Notably, CAP-induced [Ca^2+^]_i_ rises were dose-dependently inhibited by RTG (1–10 µM; Figure 3E), with about 50% inhibition of the maximal response achieved at the concentration of 1 µM; the simultaneous exposure to the Kv7 blocker XE991 fully abolished 10 µM RTG-induced inhibition of CAP-triggered [Ca^2+^]_i_ responses (Figure 3E). Notably, CAP-induced [Ca^2+^]_i_ rises were also blocked by a similar extent by 3 µM ICA-27243 (Figure 3C–E) and 10 µM (S)-1 (Figure 3D–E).

As anticipated, in the presently-described experiments, different cells were exposed to CAP or to CAP+Kv7 modulators; therefore, given that only a fraction (30/72; 40%) of F11 cells responded to CAP in control conditions, the possibility exists that the Kv7 modulators influenced the percentage of CAP-responsive cells. However, this hypothesis seems unlikely since a similar percentage of F11 cells responded to CAP when exposed to RTG (17/43, 40%), ICA-27243 (17/41, 41%), and (S)-1 (20/52 (38%), respectively.

## 3. Discussion

Neuropathic pain, caused by a lesion or disease affecting the somatosensory nervous system, has a considerable impact on patients’ quality of life, and is associated with a high economic burden on the individual and society, affecting 7–10% of the general population [40]. Peripheral neuropathic pain is due to altered function and sensitization of neurons within the peripheral nociceptive system (i.e., nociceptive neurons), the sensory system responsible for the perception of pain and the transduction of pain signals to the spinal cord [41]. At each of these sites along the various components of nociceptive pathways, neuronal excitability is controlled by a myriad of ion channels, each with an exquisite distribution pattern and specific functional properties. In particular, changes in expression and/or function of Kv7 channels in sensory neurons play a critical pathogenetic role in the development of pain states accompanying nerve crushing, inflammatory states, and central and/or peripheral neuropathies [15,16]. In addition, a functional interaction has been demonstrated between Kv7 channels and transient receptor potential vanilloid 1 (TRPV1) channels [24], expressed predominantly in peripheral nociceptors [25] and activated by TRPV1-mediated pain-inducing substances such as the endogenous inflammatory mediator BK or the exogenous burning agent capsaicin (CAP); however, whether Kv7 channels of specific molecular composition provide a dominant contribution in BK- or CAP-evoked responses is yet unknown.

The results of the present study revealed that transcripts for all five members of the Kv7 family could be detected in F11 cells, a hybrid cell line obtained from the fusion of differentiated mouse neuroblastoma cells and primary cells from rat dorsal root ganglia which has been widely used to investigate the molecular mechanisms triggered by nociceptive stimuli [31,33,34,42] and which is known to express functional TRPV1 channels [27]. Exposure of F11 cells to differentiating experimental conditions caused a substantial increase in the expression of Kv7.2, Kv7.3, and particularly Kv7.4. Patch-clamp recordings also allowed to detect a slowly-deactivating current which was potentiated or inhibited, respectively, upon exposure to Kv7 activators or blockers, strongly suggesting that the recorded current corresponded to Kv7-mediated I_KM_.

Bradykinin is a well-known endogenously-produced pain-inducing substance; among the mechanisms by which BK induces hyperalgesia are TRPV1 sensitization and inhibition of M-type K^+^ channels. Both these effects participate to BK-induced hyperexcitability of nociceptive neurons and facilitation of ascending nociceptive signals. In differentiated F11 cells, BK reversibly triggers transient increases in [Ca^2+^]_i_; in primary afferent DRG neurons, these transients largely depend on phospholipase C (PLC) activation causing TRPV1 channels sensitization [43], as well as on Ca^2+^-dependent inhibition of M-type K^+^ channels and opening of Ca^2+^-activated Cl^–^ channels [20]. In agreement with these hypotheses, BK-induced responses were significantly blunted in F11 cells by incubation in Ca^2+^-free solutions or by the canonical TRPV1 antagonist capsazepine [27]. BK-induced [Ca^2+^]_i_ increases were dose-dependently reduced by the pan-Kv7 opener RTG, with an IC_50_ of 1.04 ± 0.47 µM, a value close to the IC_50_s measured for RTG-induced activation of heterologously expressed Kv7 subunits and on native I_KM_ [44]; these results are in close agreement with the marked ability of RTG to attenuate nocifensive behavior triggered by intraplantar injection of BK in rats [20].

In the same cellular model, ICA-27243 [28], a benzamide derivative showing antiepileptic activity in a broad range of rodent seizure models and provided of a certain degree of selectivity for Kv7.2/Kv7.3 channels [37], was also investigated; notably, while RTG activates Kv7.2/Kv7.3 channels by binding to the S_5_-S_6_ pore domain, ICA-27243 binds to a pocket located in the VSD [45]. The results obtained revealed an IC_50_ of about 5 µM for ICA-27243-induced inhibition of [Ca^2+^]_i_ transients evoked by BK exposure; this value is 10 times higher than that reported on heterologously-expressed Kv7.2/Kv7.3 channels, being instead in the range of that for ICA-27243-induced activation of Kv7.4 currents [28,37,45].

On the other hand, the rather selective Kv7.4/Kv7.5 activator (S)-1 [29], an orally bioavailable acrylamide derivative showing activity in a rat cortical spreading depression model of human migraine [46] and which binds to the same pore pocket used by pan-Kv7 activators [29], dose-dependently counteracted BK-induced [Ca^2+^]_i_ rises, showing a maximal efficacy identical to that of RTG and ICA-27243 (~60% maximal blockade), but an IC_50_ of about 7 µM, thus showing a potency identical to that shown by this drug for the activation of Kv7.4-mediated currents [29]. Thus, although the selectivity of both compounds among Kv7 subunits is only relative, the present data suggest a possible, previously-unrecognized, contribution of Kv7.4 subunits in blunting BK-induced [Ca^2+^]_i_ rises.

As previously mentioned, BK-triggered nociceptor activation is mediated, at least in part, by TRPV1 activation. To investigate whether I_KM_ activators may block [Ca^2+^]_i_ rises triggered by direct-acting TRPV1 agonists, the effects of Kv7 activators in F11 cells exposed to the canonical TRPV1 agonist CAP [38] were also investigated. The results obtained showed that, similarly to BK-induced responses, CAP-induced [Ca^2+^]_i_ responses were dose-dependently inhibited by RTG, ICA-27243, and (S)-1, thus providing a strong pharmacological evidence for a critical inhibitory contribution of Kv7 subunits in the control of [Ca^2+^]_i_ responses triggered in differentiated F11 cells not only by indirect (BK), but also direct (CAP) TRPV1-activating *stimuli*. Moreover, similarly to the data obtained for BK, the rank order of potency for the presently investigated pharmacological tools (RTG > ICA-27243 > (S)-1), suggests a rank order of potency consistent with a potential role for Kv7.4 subunits in CAP-evoked [Ca^2+^]_i_ responses.

Altogether, the present results show that the pharmacological activation of neuronal Kv7 channels significantly inhibits pain-evoking cellular responses in peripheral sensory neurons, confirming the role of these channels as critical pharmacological targets for novel treatment strategies against pain states. Pharmacological tools provided of a better selectivity profile for Kv7 channels of specific molecular compositions will be needed to achieve a precise molecular identification of the molecular steps participating in nociceptor activation by endogenous and/or exogenous pain-inducing *stimuli*.

## 4. Materials and Methods

### 4.1. Cell Cultures

Cell cultures were maintained as previously described [27]. Briefly, F11 cells were grown in DMEM medium supplemented with 10% FBS, 100 U/mL penicillin/streptomycin and 2 mM l-glutamine. The cells were kept in a humidified atmosphere at 37 °C with 5% CO_2_ in 100 mm plastic Petri dishes. Differentiation of F11 cells was achieved by exposure for at least 72 h to a medium containing a lower FBS concentration (2%) and 10 µM retinoic acid [34].

For Ca^2+^ imaging and electrophysiological experiments, F11 cells were plated on glass coverslips (Carolina Biological Supply Co., Burlington, NC, USA) coated with poly-l-lysine (Sigma, Milan, Italy).

### 4.2. RNA Extraction and Semiquantitative PCR

Total RNA was isolated from undifferentiated or differentiated F11 cells using TRI-Reagent (Sigma-Aldrich, Milan, Italy), following manufacturer’s instructions. RNA samples were treated with 0.1 U/mL DNase-I (Sigma-Aldrich) for 15 min at room temperature (RT; 20–22 °C). Final preparation of RNA was considered DNA- and protein-free if the OD values ratio at 260/280 nm was >1.7. cDNA was synthesized by reverse transcription using 1–5 µg of isolated RNA as a template, 2.5 U/mL of MuLV high-capacity reverse transcriptase (Applied Biosystem, Monza, Italy) in a buffer containing 4 mM dNTP mix, 2.5 mM Random Primers, 1 U/mL RNase Inhibitor at 37 °C for 120 min. After MuLV reverse transcriptase inactivation (10 min incubation at 95 °C), the cDNA obtained was amplified in PCR gold buffer, containing 1.5 mM MgCl_2_, 0.8 mM dNTP mix, 0.5–1 mM forward and reverse primers (designed on highly-conserved sequences between mouse and rat Kv7 isoforms; [47]) and 0.1–0.25 U/mL AmpliTaq Gold (Applied Biosystem, Monza, Italy). The PCR amplification protocol was: denaturation at 95 °C for 1 min, annealing at 52 °C for 1 min, elongation at 72 °C for 1 min (30–35 cycles). To exclude the presence of genomic DNA, PCR amplification was also performed on RNA samples in which cDNA was omitted. PCR products were analyzed by electrophoretic separation on 2% agarose gel in 0.5% TBE. Acquisition and analysis of images was performed on a Gel DOC XR System (Bio-Rad laboratories, Hercules, CA, USA).

### 4.3. Quantitative Reverse-Transcription PCR (qRT-PCR)

cDNA was analyzed by qRT-PCR with the SYBR Green PCR Master Mix (Bio-Rad Laboratories Inc., Hercules, CA, USA). The qRT-PCR was carried out in a Mastercycler ep Realplex (Eppendorf, Milan, Italy). Relative mRNA expression was determined by the ΔΔC*t* method [47] using GAPDH as endogenous control. Serial cDNA dilutions were analyzed to ensure the linearity of the PCR reaction and to evaluate its efficiency.

### 4.4. [Ca^2+^]_i_ Measurements

Measurements of [Ca^2+^]_i_ were performed as previously reported [27]. Briefly, F11 cells were plated on glass coverslips and loaded with 3 mM Fura-2 acetoxymethyl ester (Fura-2 AM) for 1 h at RT in darkness in a standard solution containing (in mM): 160 NaCl, 5.5 KCl, 1.5 CaCl_2_, 1.2 MgSO_4_, 10 HEPES, 10 glucose, pH 7.4 adjusted with NaOH. Thereafter, the coverslips were washed twice with PBS to remove extracellular dye and placed in a perfusion chamber onto the stage of an inverted Leica DM IRB fluorescence microscope equipped with a 40× oil objective lens. Cells were perfused throughout the experiments with a medium of the above-mentioned composition with or without specific drugs, as specified in each experiment. Fluorescence images were acquired using a digital imaging system, composed of a cool-SNAP ES camera (Roper Scientific, Ottobrunn, Germany), DeltaRAM XTM Microscope Illuminator (Photon Technology International, Birmingham, NJ, USA) and MetaFluor Imaging System software (Molecular Device, Sunnyvale, CA, USA). Cells were alternatively illuminated at wavelengths of 340 nm and 380 nm by a 100 W Xenon lamp; the emitted light was passed through a 512-nm barrier filter. Fura-2 fluorescence was recorded every 3–4 s, and fluorescence intensity values converted in Ca^2+^ concentrations assuming a K_d_ of 224 nM [48]. In each experiment, background fluorescence was recorded in a field devoid of cells and subtracted from the measured emission of each channel. Only cells with basal [Ca^2+^]_i_ in the range of 90–120 nM were analyzed.

### 4.5. Whole-cell Electrophysiology

Currents from differentiated F11 cells were recorded by perforated whole-cell recordings (using nystatin) at RT using an Axopatch 200A amplifier (Molecular Devices, Union City, CA, USA) with glass micropipettes of 3–5 MΩ resistance. The extracellular solution contained (in mM): 138 NaCl, 2 CaCl_2_, 5.4 KCl, 1 MgCl_2_, 10 glucose, and 10 HEPES, pH 7.4 adjusted with NaOH. The pipette (intracellular) solution contained (mM): 140 KCl, 2 MgCl_2_, 10 EGTA, 10 HEPES, 5 Mg-ATP, pH 7.4 adjusted with KOH. pCLAMP software (version 10.2; Molecular Devices, Union City, CA, USA) was used for data acquisition and analysis. Currents were leak subtracted off-line; currents recorded in the presence of indicated drugs were normalized to those measured from the same cell in control solution.

### 4.6. Statistics

Data are expressed as the mean ± SEM. Statistically significant differences between the data (*p* < 0.05) were evaluated with the Student’s t-test or by the ANOVA, when multiple groups were compared, by using STAT software, version 1.0 (author Glantz SA).
